# Predicting curve progression for adolescent idiopathic scoliosis using random forest model

**DOI:** 10.1371/journal.pone.0273002

**Published:** 2022-08-11

**Authors:** Ausilah Alfraihat, Amer F. Samdani, Sriram Balasubramanian

**Affiliations:** 1 School of Biomedical Engineering, Science and Health Systems, Drexel University, Philadelphia, Pennsylvania, United States of America; 2 Shriners Hospitals for Children, Philadelphia, Pennsylvania, United States of America; University of California San Francisco, UNITED STATES

## Abstract

**Background:**

Adolescent Idiopathic Scoliosis (AIS) is a three-dimensional (3D) spinal deformity characterized by coronal curvature and rotational deformity. Predicting curve progression is important for the selection and timing of treatment. Although there is a consensus in the literature regarding prognostic factors associated with curve progression, the order of importance, as well as the combination of factors that are most predictive of curve progression is unknown.

**Objectives:**

(1) create an ordered list of prognostic factors that most contribute to curve progression, and (2) develop and validate a Machine Learning (ML) model to predict the final major Cobb angle in AIS patients.

**Methods:**

193 AIS patients were selected for the current study. Preoperative PA, lateral and lateral bending radiographs were retrospectively obtained from the Shriners Hospitals for Children. Demographic and radiographic features, previously reported to be associated with curve progression, were collected. Sequential Backward Floating Selection (SBFS) was used to select a subset of the most predictive features. Based on the performance of several machine learning methods, a Random Forest (RF) regressor model was used to provide the importance rank of prognostic features and to predict the final major Cobb angle.

**Results:**

The seven most predictive prognostic features in the order of importance were initial major Cobb angle, flexibility, initial lumbar lordosis angle, initial thoracic kyphosis angle, age at last visit, number of levels involved, and Risser "+" stage at the first visit. The RF model predicted the final major Cobb angle with a Mean Absolute Error (MAE) of 4.64 degrees.

**Conclusion:**

A RF model was developed and validated to identify the most important prognostic features for curve progression and predict the final major Cobb angle. It is possible to predict the final major Cobb angle value within 5 degrees error from 2D radiographic features. Such methods could be directly applied to guide intervention timing and optimization for AIS treatment.

## 1. Introduction

Adolescent Idiopathic Scoliosis (AIS) is a three-dimensional (3D) spinal deformity characterized by adolescent-onset, coronal curvature, and rotational deformity. AIS is the most common form of spinal deformity, accounting for 80% of pediatric scoliosis and affects 2–4% of children throughout their growth spurt [1, 2]. The gold standard to quantify the severity of AIS is by Cobb angle measurement from radiographs, which is the angle between the two most tilted vertebrae in the spinal curvature [3]. AIS has a heterogeneous natural history, with some patients presenting with rapidly progressive curves and others progressing slowly [4–6]. Mild to moderate progressive scoliosis may result in cosmetic deformity, back pain, functional impairments, and psychological issues [7, 8], whereas severe cases are associated with cardiac dysfunction and pulmonary constraints [9–12]. Treatment of AIS includes braces and surgical interventions. Bracing is recommended for AIS patients with a curvature of 25–45 degrees [2]. Surgery is considered in skeletally immature patients with a structural thoracic curve greater than 45 degrees or patients with continued progression [13].

The prediction of curve progression is important for treatment selection and timing. A major concern of orthopedic surgeons in managing AIS patients with a minor curvature is identifying which curves will progress to moderate or severe deformities that will require surgical intervention [14–16]. As the progression of scoliosis deformity is primarily dependent on growth during skeletal immaturity [17–19], predicting the risk of curve progression relies on surrogate indicators. Previous studies have associated various clinical and radiographic factors such as gender, age, curve pattern, curve magnitude, apical level location, flexibility, biomarkers, and skeletal maturity with curve progression [17, 20–32]. The patient’s younger chronological and skeletal ages at curve onset, greater initial spine curvature, and delayed onset of menarche are known factors associated with increased curve progression [23, 26–28, 33]. Thoracic curves are more likely to progress than lumbar curves because of the inherent stiffness associated with the presence of the rib cage [30, 31], and double major curves are more likely to progress than single curves [23, 34]. Wedging of the intervertebral disc and adjacent vertebrae are also factors associated with severe spine curvature [5, 24, 29, 35–37]. However, attempts to use each of these features individually as a prognostic factor for curve progression showed inaccurate (i.e., a high number of false positive and false negative) predictions [22]. Although there is a consensus in the literature regarding prognostic factors associated with curve progression, the order of importance, as well as the combination of factors that are most predictive of curve progression is unknown.

Statistical modeling has been used to predict the progression of curves at risk for surgical intervention. Sitoula et al. found that Sanders skeletal maturity and initial major Cobb angle were correlated to curve progression [28]. They proposed a logistic regression model which predicted the probability of curve progression beyond 50 degrees in AIS patients, a threshold typically suggested for surgery. Zhang et al. formulated a multivariate logistic regression model comprising clinical parameters and biomarkers to evaluate the probability of curve progression to a Cobb angle greater than 40 degrees [38]. However, logistic regression models assume a linear relationship between the features and the response, while spine growth and curve progression are non-linear processes [39]. Recent studies have also attempted to predict curve progression using Machine Learning (ML) algorithms [40–43]. ML refers to the capability of data-driven algorithms to gain knowledge regarding a system directly from data without predetermining mechanistic relationships that rule the system [44]. Moreover, ML algorithms are self-learning and therefore, able to adaptively improve their predictive performance with each new data sample to detect hidden patterns in high dimensional and heterogeneous data [44]. Deng et al. used a random forest model to predict the final major Cobb angle in AIS patients and suggested a method to handle missing values; however, this model was based on single curves, and therefore, it is not applicable to double and triple curves [45]. Wu et al. merged fuzzy c-means clustering methods and artificial neural network (ANN) to predict the future scoliosis Cobb angle [40]. Scoliosis patients (n = 61) were included with at least four follow-up Cobb angle measurements. However, their model may not be applicable to new patients or those with fewer records. Chalmers et al. proposed a conditional fuzzy clustering model to predict curve progression in braced AIS patients [41, 42]. Although the model achieved good performance in clustering, the importance of features and their relationships may not be properly identified. Ajemba et al. utilized common indicators of AIS patients to predict the progression of scoliosis with support vector machine [43]. However, their study used a small sample size (n = 44), and the results had low accuracy (65%). Moreover, Edgar et al. used statistical generative model to predict the shape variation of the spinal curve. However, 3D reconstructions of the spinal curve from 2D radiographs are sophisticated [6, 46–48].

Considering that AIS is a multifactorial pathology, the predictability of progression remains challenging. Hence, the objectives of the current study are to (1) create an ordered list of prognostic factors (features) that most contribute to curve progression using backward elimination feature selection method and (2) develop and validate an interpretable Machine Learning (ML) model to predict final major Cobb angle in AIS patients. We hypothesize that there is a combination of clinical features that significantly contribute to curve progression, and a ML model that uses these features can provide accurate and robust curve progression prediction, provided sufficient patient demographic and radiographic data.

## 2. Methods

### 2.1. Inclusion criteria for patients selection

After institutional review board approval (Protocoal# PHL1904R) was obtained by the western institutional review board, pediatric patients from the Shriners hospitals for children database (1995–2019) were retrospectively selected based on the following inclusion criteria: 1) primary diagnosis of AIS with any Lenke type, 2) males and females ages 10–19 years, and 3) at least two follow-up visits with preoperative (posteroanterior (PA) or anteroposterior (AP), lateral and side bending) radiographic images (X-ray, CT or EOS-imaging). Before accessing the database, all data were fully anonymized, and as the data is retrospective, the western institutional review board waived the requirement for informed consent.

### 2.2. Data collection

Previously reported demographic and radiographic features associated with curve progression were collected. In the current study, eight continuous and six categorical features were used (Tables 1 and 2).

**Table 1:** Description of continuous features.

Measurement	Mean ± STD	Range
Initial major Cobb angle (°)	48.9±13.9	(10.9–99.5)
Initial kyphosis angle T2-T12 (°)	24.2±10.9	(0–51.3)
Initial lumbar lordosis angle L1-L5 (°)	53.6±12.1	(3.4–87.1)
Age at first visit (Years)	12.7±1.7	(10–17.8)
Age at last visit (Years)	13.6±1.6	(10.4–18.6)
Apex wedge angle (°)	56.7±4.1	(0–21)
Time span (Years)	0.9±1	(0.08–6.5)
Flexibility (%)	57±19.3	(0.05–0.99)
Apex axial rotation (°)	20.9±7.9	(0.02–41.6)
Final major Cobb angle (°)	59.4±12.4	(35.7–107)

**Table 2:** Description of categorical features.

Feature	Frequency (i.e., number of patients)									
Gender	Female					Male				
	169					24				
Lenke type	1	2			3	4	5			6
	116	11			38	1	15			12
Brace status	Brace					No Brace				
	143					50				
Apex location	T6	T7	T8	T9	T10	T11	T12	L1	L2	L3
	3	14	59	61	17	7	8	14	9	1
Number of levels involved	4	5	6		7	8	9	10		
	2	7	44		76	44	12	8		
Risser "+" stage at the first visit	0-	0+	1		2	3	3/4	4		5
	74	38	10		14	13	12	16		16

#### 2.2.1. Demographic features

Based on the inclusion criteria, 193 patients (24 males, 169 females) were selected for the current study. Gender, age, and bracing status at initial and final follow-up visits for each subject are shown in Tables 1 and 2.

#### 2.2.2. Radiographic features

**Fig 1:**
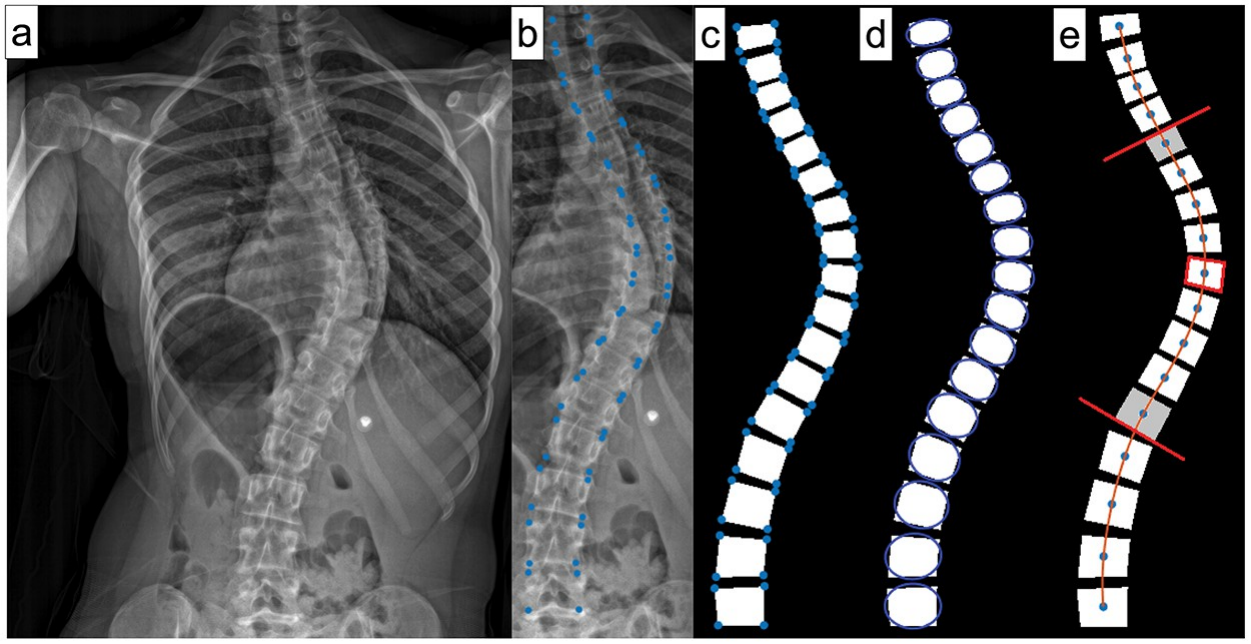
Steps of Cobb angle measurement from frontal radiographs. a. Plain frontal X-ray b. Four LMPs were selected per vertebra c. Polygon fit through each vertebra vertices (LMPs) d. Best fit ellipse through each polygon to calculate the orientation of each vertebra as the angle between the major axis of the ellipse and the horizontal e. A cubic spline was fit through the centroids of the vertebrae, the most tilted vertebrae above and below the apical level were identified, and the Cobb angle was calculated between these vertebrae.

For all patients, preoperative standing (PA or AP, and lateral) and side bending spinal radiographs, including the pelvis, were collected. Custom script (MATLAB, The MathWorks Inc, Natick, MA) was used to select four landmark points (LMPs) on the vertices of each vertebra in standing and side bending radiographs (Fig 1a and 1b). Using these LMPs, the following features were extracted at initial and final follow-up visits: Cobb angle, vertebral wedge angle, flexibility, apical level, and number of levels involved in the curve. Thoracic Kyphosis (T2-T12) and lumbar lordosis (L1-L5) angles were measured manually from lateral radiographs. Axial rotation of the apical level was measured in the frontal radiograph based on Raimondi’s method [49, 50].

Using a custom script, polygons were fit through the LMPs to represent each vertebra’s boundaries and their centroids (Fig 1c). Additionally, an ellipse was fit through each polygon, and the orientation of the vertebra as measured by the angle of the major axis with respect to the horizontal was calculated (Fig 1d). A cubic spline was fit using the centroids, and the maxima and minima points were identified. The apical level was defined as the closest centroid from the maxima or the minima, depending on the sidedness and location of the curve. The most tilted vertebrae above and below the apical level were identified, and the Cobb angle was calculated between these vertebrae. The apical vertebral wedge angle was calculated as the angle between the superior and inferior endplates of the apical vertebra (Fig 1e).

**Fig 2:**
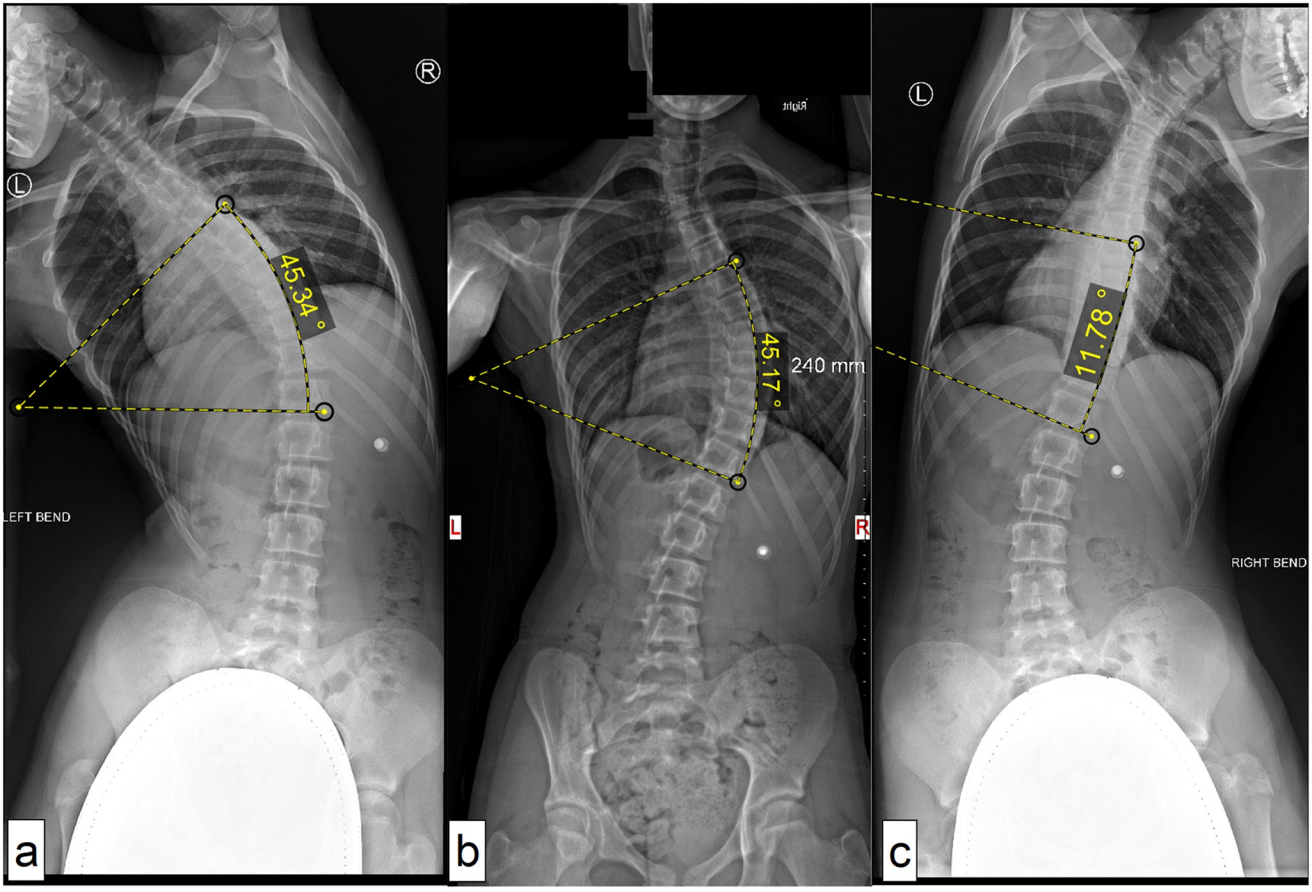
Exemplar flexibility measurement.

As side bending radiographs were not available for some patients, modified Lenke (mLenke) classification system was used instead of the original Lenke system [28, 51]. The major curve was defined as that with the greatest Cobb angle, and the minor curve with a Cobb angle ≥ 80% of the major curve magnitude was defined as structural. For the mLenke classification, six curve types were used: type 1 (main thoracic), type 2 (double thoracic), type 3 (double major; thoracic curve > lumbar curve), type 4 (triple major), type 5 (thoracolumbar/lumbar), and type 6 (double major; thoracolumbar or lumbar curve > thoracic curve). The flexibility was assessed using the rate of difference in Cobb angle between the standing (PA or AP) and side bending radiographs [52] (Fig 2).

The Risser stage, a widely used indicator of skeletal maturity [53], describes the ossification patterns and fusion of the iliac apophysis [53, 54]. However, the Risser staging system does not predict the growth spurt as the iliac apophysis starts at Risser stage 1. Therefore, its accuracy has been contested [28, 55]. In the current study, an eight-point Risser "+" staging was used as a more precise and detailed marker of skeletal maturity during growth spurt [55]. The Risser "+" system combines the North American (NA) and European (EU) versions of the traditional Risser score [55]. The Risser "+" system consists of grade: 0-(open triradiate cartilage (TRC)), 0+ (Closed TRC), 1 indicates 0–25% coverage, 2 indicates 25%-50% coverage, 3 indicates 50%-75% coverage, 3/4 indicates 75%-100% coverage, 4 indicates start of fusion and 5 indicates complete fusion.

### 2.3. Sequential Backward Floating Selection (SBFS)

To improve prediction accuracy and ease of data interpretation, as well as to reduce model complexity and eliminate correlated or redundant features, Sequential Backward Floating Selection (SBFS) method was used. SBFS was implemented using mlxtend (version 0.18.0 For python 3.6) [56] and was used as part of a pipeline including Scikit-learn’s (version 0.21.3 for python 3.6) [57] random forest and cross-validation. SBFS starts with the whole feature set as input (Table 3) and performs regression between the features and the target (final major Cobb angle). Based on the regression performance metric (i.e., Mean Absolute Error (MAE)), the feature that minimizes the performance metric upon removal is excluded, and the model is refitted. After each removal step, there is an additional conditional inclusion step to add features after they were excluded, for which the MAE is minimized. The subset of features with the minimum MAE was selected as input for the predictive model.

**Table 3:** Indices of input features to RF model.

Index	Feature
0	Initial lumbar lordosis angle
1	Initial thoracic kyphosis angle
2	Age at the first visit
3	Age at last visit
4	Time-span
5	Apex wedge angle
6	Lenke type
7	Flexibility
8	Apex axial rotation
9	Initial major Cobb angle
10	Brace status
11	Gender
12	Number of levels involved
13	Apex location
14	Risser "+" stage at the first visit

### 2.4. Machine learning model selection and optimization

The dataset of the most predictive features (for all patients) was randomly split into training (75%) and testing (25%) datasets. Using scikit-learn (version 0.21.3 for python 3.6) [57], three commonly used machine learning models namely, Random Forest (RF) [58], Support Vector Machine (SVM) [59, 60], and ANN [61, 62] were trained using 5-fold cross-validation and tested to predict final major Cobb angle.

To optimize the models’ performance and avoid overfitting, ranges of hyperparameters were defined and tuned using Scikit-Learn’s GridSearchCV (version 0.21.3 for python 3.6) [57]. Based on a 5-fold cross-validation, the combination of hyperparameters that produced the best performance metric (lowest MAE) was selected for each model.

### 2.5. Performance evaluation metric

The performance of the ML models was evaluated with Mean Absolute Error (MAE). MAE is the average of the absolute differences (i.e., residuals) between the final major Cobb angle magnitude predicted by the ML model and the actual final major Cobb angle value (Eq 1). An MAE of 5 degrees or less was considered clinically acceptable based on measurement error of medical imaging data [63, 64].


$$MAE=\frac{1}{n}\sum_{j=1}^{n}\left|y_{j}-{\hat{y}}_{j}\right|$$ (1)

### 2.6. Deployment of the curve progression prediction model

**Fig 3:**
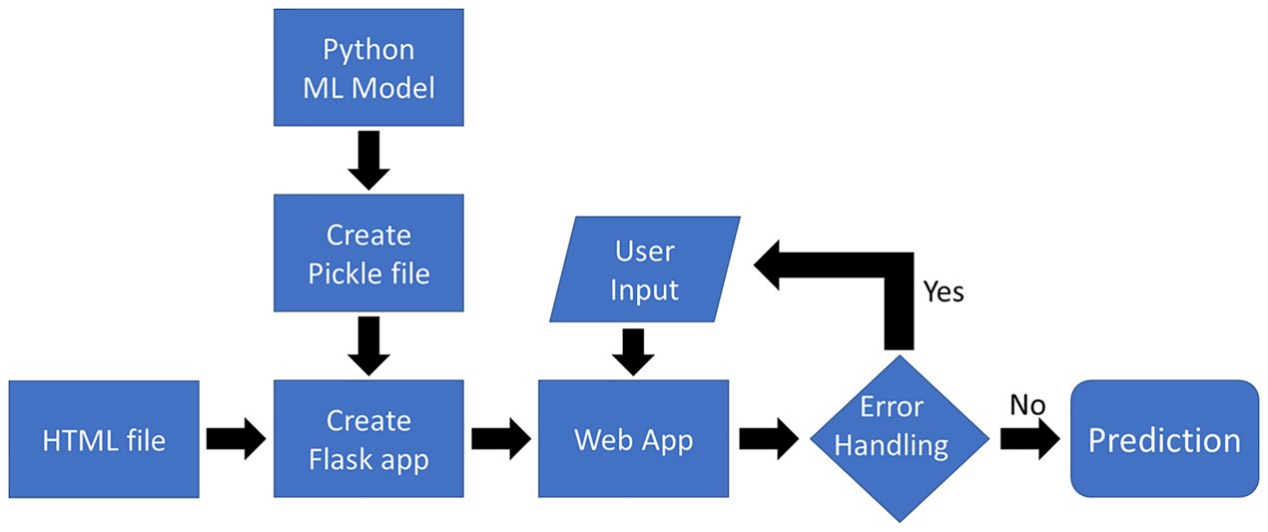
Flowchart of the curve progression model deployment.

To ensure the clinical translation of the reported findings, and for the model to be more widely used, a web user-interface app to predict the final Cobb angle in AIS patients was created. Model deployment was done using Flask and Heroku (Fig 3), where Flask is a micro web framework to create web applications, and Heroku is a cloud platform to host such web applications.

## 3. Results

Continuous features (n = 9): includes initial major Cobb angle, initial lumbar lordosis angle, initial thoracic kyphosis angle, age at initial follow-up, age at final follow-up, initial apical wedge angle, the time span between age at initial and final follow-ups, flexibility and axial rotation. Data of the continuous features at the initial visit are shown in Table 1.

Categorical features (n = 6) include three nominal features, namely Lenke type, gender, and brace status, and three ordinal features: apex location, number of levels involved in the curve, and Risser "+" stage. Categorical features were encoded. The description of the categorical features is shown in Table 2.

### 3.1. Most predictive features selected from the SBFS method

The input features to the SBFS and their corresponding indices are listed in Table 3. Table 4 shows a descending order of the features subsets as input to the RF model based on their corresponding 5-fold cross-validation average MAE. The best seven predictive features out of the fifteen features selected by SBFS are: initial major Cobb angle, flexibility, initial lumbar lordosis angle, initial thoracic kyphosis angle, age at last visit, number of levels involved, and Risser "+" stage at the first visit.

**Table 4:** Output from SBFS ranked in descending order.

Number of features	Features Indices	5-fold cross-validation Average MAE scores (Standard Deviation)	Confidence Interval (CI) bound
**7**	**(0, 1, 3, 7, 9, 12, 14)**	**6.146 (0.97)**	**1.25**
6	(0, 3, 7, 9, 12, 14)	6.180 (0.97)	1.25
8	(1, 2, 3, 4, 7, 9, 12, 14)	6.188 (0.95)	1.23
9	(1, 2, 3, 4, 6, 7, 9, 12, 14)	6.193 (1.04)	1.33
6	(3, 7, 9, 12, 14)	6.194 (0.97)	1.25
10	(0, 1, 2, 3, 4, 6, 7, 9, 12, 14)	6.201 (1.11)	1.42
4	(7, 9, 12, 14)	6.220 (1.01)	1.30
11	(0, 1, 2, 3, 4, 5, 6, 7, 9, 12, 14)	6.224 (1.10)	1.42
12	(0, 1, 2, 3, 4, 5, 6, 7, 9, 12, 13, 14)	6.261 (1.11)	1.42
3	(9, 12, 14)	6.280 (0.95)	1.22
13	(0, 1, 2, 3, 4, 5, 6, 7, 9, 10, 12, 13, 14)	6.296 (1.08)	1.39
2	(9, 14)	6.381 (0.87)	1.12
14	(0, 1, 2, 3, 4, 5, 6, 7, 8, 9, 10, 12, 13, 14)	6.390 (1.21)	1.55
15	(0, 1, 2, 3, 4, 5, 6, 7, 8, 9, 10, 11, 12, 13, 14)	6.557 (1.30)	1.67
1	9	6.807 (0.79)	1.01

MAE of final major Cobb angle prediction, CI represents the 95% confidence interval around the computed cross-validation scores.

### 3.2. Random forest regressor to predict final major Cobb angle

**Fig 4:**
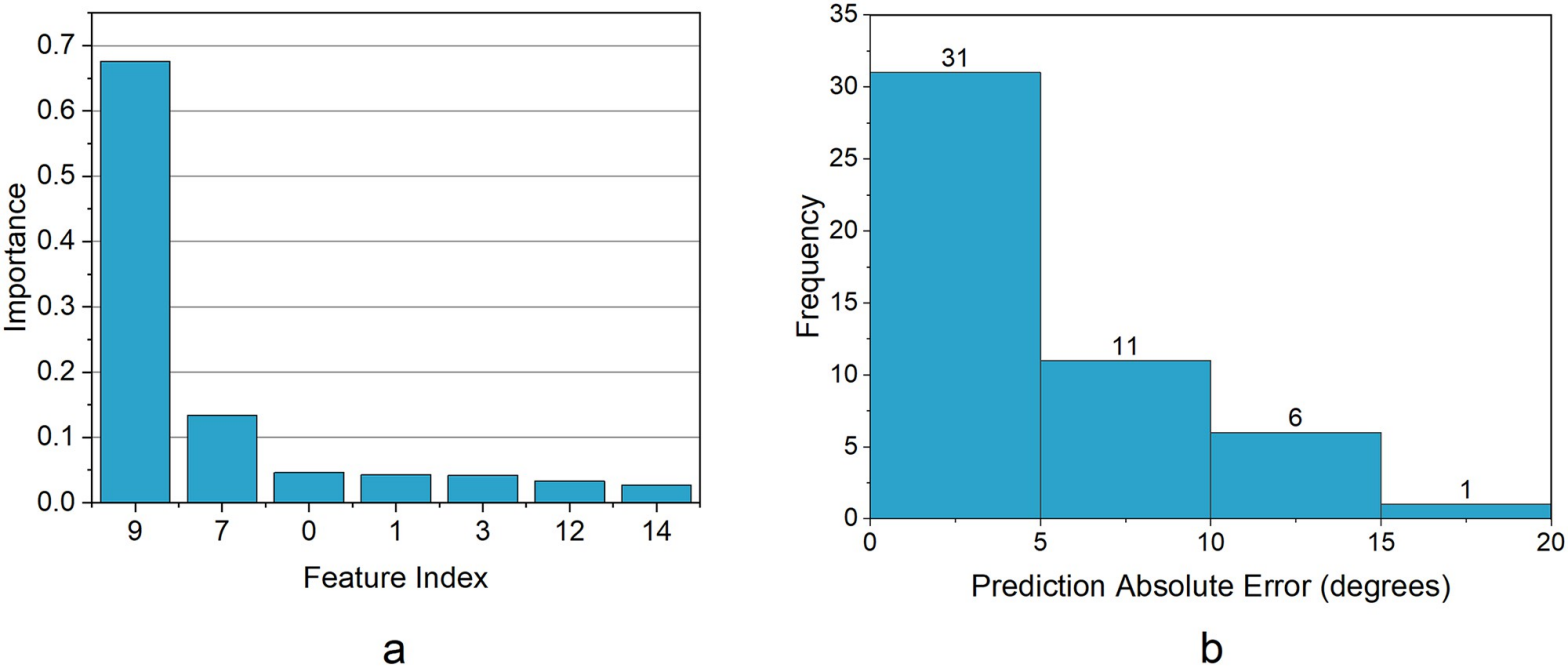
a. Rank of feature importance of most predictive features b. Frequency of prediction error of the testing dataset.

The range of hyperparameters and the selected values used to optimize the ML models are shown in Table 5. The seven most predictive features selected by the SBFS algorithm were used as inputs for the ML models. Based on its superlative performance (Table 6), the RF model was selected as the ML method to predict the final major Cobb angle. The rank of the importance of the prognostic features and their respective weights are shown in Fig 4a and Table 7, respectively. The testing errors and their frequencies are shown in Fig 4b.

**Table 5:** Hyperparameters grid values and the selected values for optimized models.

Hyperparameter	Range of values	Selected Value
Random Forest		
n_estimators	[100–500]	291
max_features	[1–7]	5
max_depth	[1–50]	30
min_samples_split	[2–11]	7
min_samples_leaf	[1–11]	2
Support Vector Machine		
Epsilon	[0,1,10,100,1000]	0
Gamma	[1,0.1,0.001,0.0001]	0.001
Artificial Neural Network		
batch_size	[10,20,30,40,50]	10
epochs	[10,20,30,40,50]	50
Optimizer	[’adam’, ’rmsprop’]	rmsprop

**Table 6:** Performance of the ML models in terms of Mean Absolute Error (MAE).

Model	5-Fold cross validation Training	Testing
Random Forest	3.54	4.64
Gradient Boosting Regressor	3.72	4.79
Support Vector Machine	6.14	6.84
Artificial Neural Network	4.77	5.82

**Table 7:** Rank and weights of most important features to predict curve progression.

Rank	Feature	Importance (Weight)
1	Initial major Cobb angle	0.676
2	Flexibility	0.134
3	Initial lumbar lordosis angle	0.046
4	Initial thoracic kyphosis angle	0.043
5	Age at last visit	0.042
6	Number of levels involved	0.033
7	Risser "+" stage at the first visit	0.027

### 3.3. Web user-interface app for AIS curve progression prediction

The web user-interface app is publicly available at: https://biomed.drexel.edu/labs/obl/toolkits/predict-curve-progression/

## 4. Discussion

This is the first study to apply machine learning methods to longitudinal data from a cohort of AIS patients to identify a rank-ordered list of the most important features associated with curve progression. An RF model was developed and validated using these patient-specific prognostic features (i.e., demographic and radiographic measurements) to predict curve progression in AIS patients. The composite model presented in the current study is well suited to characterize the curve progression of a multifactorial AIS pathomechanism.

Previous studies that reported prognostic features associated with curve progression had several limitations. While Wu et al. predicted follow-up Cobb angle based on a single feature (current Cobb angle), radiographs at multiple longitudinal timepoints were needed [40]. The predictive model for curve progression in braced AIS patients developed by Chalmers et al. [41] did not include two skeletal maturity measurements, namely Risser sign and triradiate cartilage status that were previously reported to be highly prognostic [18, 19]. In contrast, the current study used Risser "+" staging, which combines these skeletal maturity grades. Ajemba et al. [43] reduced the number of features using principal component analysis; however, the principal components are difficult to interpret. It is also important to note that none of the previously reported models provided an ordered list of the importance of predictive features.

The current study is the first to predict the magnitude of the final major Cobb angle (with a mean absolute error of 4.64 degrees) to a clinically acceptable error based on 2D radiographic features at the initial curve presentation. While Deng et al. [45] also used a RF model to predict the final major Cobb angle based on the initial major Cobb angle, bracing status, and age, the dataset had a large number of missing values which would increase the risk of model bias. The combined fuzzy c-means clustering, ANN, and cubic spline extrapolation method reported by Wu et al. predicted Cobb angle with good accuracy (mean error of 4.4±1.86 degrees) [40]. However, the model required three Cobb angle measurements at 6- to 12-month intervals and, therefore, could be used for early prognosis. Furthermore, the prediction accuracy was affected by the 3D reconstruction methods used in their study. More recently, 3D spine shape variations based on independent component analysis differed from the actual curvature by 1.83, 5.18, and 4.79 degrees of Cobb angles in the proximal thoracic, main thoracic, and thoracolumbar, lumbar sections, respectively [46]. While this method seems promising, it may be difficult to integrate into the clinical workflow due to limitations associated with sophisticated 3D spine reconstructions from biplanar radiographs. In contrast, the current method uses 2D radiographic features that can be easily measured during routine clinical care.

The current study reports that thoracic kyphosis and lumbar lordosis are important prognostic factors in predicting scoliotic curve progression. Although it might not be obvious that sagittal plane deformity (i.e., kyphosis and lordosis) can be predictive of deformity progression in the coronal plane (i.e., scoliosis), previous studies have reported asymmetric alterations in loading due to coupled motion patterns in the scoliotic spine [65]. Coupled rotation and lateral bending motions, while forcing the posterior elements towards the curve’s concavity [65–68], create biplanar asymmetry [65, 68, 69]. Therefore, the structural alterations in the coronal plane are a coupled effect that accompanies the culmination of a sagittal plane deformity and a transverse plane rotation. Such scoliotic deformity progression is a result of a vicious cycle, according to the Heuter-Volkmann principle [70, 71].

DNA-based prognostic tests to predict curve progression in AIS have gained interest since the first genome-wide association studies (GWAS) [72]. Various single nucleotide polymorphisms (SNP) have been reported in several cohorts comparing AIS and control populations [73, 74]. The majority of the associations reported previously are not replicable in different ethnic groups, and their biological roles in AIS remain unclear [75]. A saliva-based genetic prognostic test called ScolioScore analyzes 53 SNPs to identify patients’ risk of AIS curve progression [76]; however, its predictive value is limited [76, 77]. Follow-up replication studies conducted by Ogura [78] and Tang [79] could not confirm the association between the 53 SNPs and curve progression in Japanese and French-Canadian populations, respectively. Due to the aforementioned limitations and since DNA-based testing results were unavailable for the AIS patients in the current study, gene-based features were not included.

The proposed prediction model outperformed existing models in terms of higher accuracy, interpretability, greater sample size, which prevents overfitting, and ease of use, utilizing features that are routine clinical measurements and relatively easy to extract rather than sophisticated 3D shape reconstruction from 2D radiographs. This study has a few limitations: Although RF is a robust regression algorithm, it is unable to discover trends that would enable it to extrapolate values that fall outside the range of the features in the training set. However, this limitation can be reduced by increasing the size of the training set. Moreover, the outcomes of this study may be affected by institutional bias, and future work comparing the results on expanded datasets from other institutions may be beneficial. In addition, because of the retrospective nature of this study, data on menarche status, bracing onset, brace compliance, and brace duration were not available. Also, Sanders skeletal maturity [27] was not used since not all patients had hand radiographs. Menarche status, bracing, and Sanders skeletal maturity can be evaluated as predictive features for future prospective studies.

## 5. Conclusions and future work

In this study, a machine learning model (Random Forest method) was developed and validated to predict the final major Cobb angle in AIS patients. The results indicate that it is possible to predict the value of the final major Cobb angle with an accuracy of 5 degrees as compared to 2D radiographic measurements. A web user-interface app for AIS curve progression prediction was also created. This web user-interface app could be used by clinicians to help guide the timing and optimization of AIS treatment methods. Also, this app can be used to educate patients and their families about patient-specific spine deformity characteristics, risk of progression, and possible treatment options.
